# How to quantify exposure to traumatic stress? Reliability and predictive validity of measures for cumulative trauma exposure in a post-conflict population

**DOI:** 10.3402/ejpt.v6.28306

**Published:** 2015-11-19

**Authors:** Sarah Wilker, Anett Pfeiffer, Stephan Kolassa, Daniela Koslowski, Thomas Elbert, Iris-Tatjana Kolassa

**Affiliations:** 1 Clinical & Biological Psychology, Institute of Psychology and Education, Ulm University, Ulm, Germany; 2 Vivo International, Allensbach, Germany; 3 Clinical Psychology, University of Konstanz, Konstanz, Germany; 4 SAP Switzerland AG, Tägerwilen, Switzerland

**Keywords:** Cumulative trauma exposure, event list, reliability, predictive validity, posttraumatic stress disorder

## Abstract

**Background:**

While studies with survivors of single traumatic experiences highlight individual response variation following trauma, research from conflict regions shows that almost everyone develops posttraumatic stress disorder (PTSD) if trauma exposure reaches extreme levels. Therefore, evaluating the effects of cumulative trauma exposure is of utmost importance in studies investigating risk factors for PTSD. Yet, little research has been devoted to evaluate how this important environmental risk factor can be best quantified.

**Methods:**

We investigated the retest reliability and predictive validity of different trauma measures in a sample of 227 Ugandan rebel war survivors. Trauma exposure was modeled as the number of traumatic event types experienced or as a score considering traumatic event frequencies. In addition, we investigated whether age at trauma exposure can be reliably measured and improves PTSD risk prediction.

**Results:**

All trauma measures showed good reliability. While prediction of lifetime PTSD was most accurate from the number of different traumatic event types experienced, inclusion of event frequencies slightly improved the prediction of current PTSD.

**Conclusions:**

As assessing the number of traumatic events experienced is the least stressful and time-consuming assessment and leads to the best prediction of lifetime PTSD, we recommend this measure for research on PTSD etiology.

In industrial countries, the lifetime prevalence of posttraumatic stress disorder (PTSD) was estimated to be below 10%, although the majority of individuals reported at least one traumatic experience (Kessler, Sonnega, Bromet, Hughes, & Nelson, 1995). These findings suggested a high variability in the psychological response to trauma and raised the interest in individual PTSD risk factors (DiGangi et al., 2013) including genetic susceptibility factors (Cornelis, Nugent, Amstadter, & Koenen, 2010).

While response variation following single trauma is high, PTSD prevalence approaches 100% at extreme levels of trauma exposure (Kolassa, Ertl, Kolassa, Onyut, & Elbert, 2010; Neuner et al., 2004). Cumulative exposure to traumatic stressors enhances PTSD risk and symptom severity in a dose-dependent manner, a phenomenon termed *building block effect* (Kolassa et al., 2010; Mollica, McInnes, Poole, & Tor, 1998; Neugebauer et al., 2009; Neuner et al., 2004).

Accordingly, investigations of individual PTSD risk factors need to consider the effect of cumulative traumatic experiences to obtain valid conclusions. For instance, it is highly recommended to include trauma exposure in genetic studies on PTSD risk and to model gene×environment interactions (Cornelis et al., 2010; Wilker & Kolassa, 2013). However, in contrast to the agreement on the necessity to include trauma exposure in etiological research on PTSD, relatively little research has been devoted on how to best quantify and assess the extent of trauma exposure (Weathers & Keane, 2007).

## Assessing the number of traumatic event types versus event frequencies

In the context of the retrospective assessment of cumulative traumatic experiences in conflict-affected populations, assessing the number of different traumatic event types experienced via event checklists has been considered to be more reliable than an assessment including the respective traumatic event frequencies. It was reasoned that many survivors had experienced a specific traumatic event type so many times that it would be difficult to report the event frequency (Neuner et al., 2004).

Studies investigating the reliability of the reported number of traumatic event types report different reliability coefficients which vary as a function of the study population and the test–retest interval. For instance, Bramsen, Dirkzwager, Van Esch, and Van der Ploeg (2001) assessed trauma exposure in a sample of 137 military veterans with a test–retest interval of 1 year, and reported a reliability of *r*=0.72. The same reliability was also reported for a sample of 309 heroin users (of which 92% reported trauma exposure at baseline) with a test–retest interval of 2 years (Mills, Teesson, Darke, & Ross, 2007). Other studies reported reliability coefficients between 0.74 and 0.93 over intervals of 1–4 weeks (Carlson et al., 2011; Goodman, Corcoran, Turner, Yuan, & Green, 1998; Gray, Litz, Hsu, & Lombardo, 2004; Hollifield et al., 2006; Mollica et al., 1992).

By contrast, reliability reports of trauma measurements which consider the frequency of the experienced events are scarce. Roemer, Litz, Orsillo, Ehlich, and Friedman (1998) investigated war trauma exposure and asked respondents to report the frequency of seven traumatic events on a 0–4 Likert scale referring to event frequencies of 0, 1–3, 4–12, 13–50, and >50. The resulting frequency score had relatively low test–retest reliability (*r*=0.66) over a period of 1–3 years in a sample of US soldiers who had served in Somalia. Strikingly, test–retest reliability over a 1-week interval of the combat exposure scale, which also assesses combat event frequency on a similar Likert scale, was 0.97 in a sample of Vietnam veterans (Keane et al., 1989). High retest reliability coefficients of self-reported frequencies of war events and atrocities in a sample of military veterans (*r*=0.83–0.87) were also observed by Unger, Gould, and Babich (1998) over a 4-week interval. Hence, the inconsistent results concerning reliability of self-reported event frequencies warrant further investigation. Furthermore, we did not find any report which addressed the question, whether the number of traumatic event types or the reported event frequencies is the more reliable measurement empirically by comparing the two trauma measures in the same population.

Next to the reliability of trauma exposure assessments, it is at least as important to evaluate the validity of the different assessment methods to measure the construct trauma load as a risk factor for PTSD. However, to our best knowledge, studies comparing different trauma exposure measurements regarding their ability to predict PTSD (i.e., predictive validity) are missing. Such investigations are urgently needed to develop standard recommendations as to how the risk factor trauma load should be assessed in etiological research on PTSD (Weathers & Keane, 2007; Wilker & Kolassa, 2013).

## Developmental timing of trauma exposure

If traumatic events are experienced during developmental sensitive periods, they are believed to have an especially strong impact on the development of childhood and adult psychopathology (Enlow, Blood, & Egeland, 2013; McLaughlin, Conron, Koenen, & Gilman, 2010; Teicher & Samson, 2013). These findings are supported by evidence that childhood, but not adult trauma, is associated with distinct epigenetic (Klengel et al., 2013; Mehta et al., 2013) and neurobiological alterations (Eckart et al., 2012). Furthermore, several gene×environment interactions in the prediction of PTSD risk were only found if childhood, but not adult trauma, was chosen as the environmental exposure variable (Binder et al., 2008; Mehta et al., 2013). Taken together, these findings raise the possibility that the assessment of the developmental timing of traumatic events could enhance the prediction of PTSD risk.

## The role of PTSD-associated memory impairments in retrospective reports of trauma exposure

A characteristic feature of PTSD are intrusive memories of the traumatic events experienced in here-and-now quality, often accompanied by difficulties to voluntarily recall their chronological order and corresponding contextual information (Brewin, 2015). The fear network model (Foa & Kozak, 1986; Kolassa & Elbert, 2007; Rockstroh & Elbert, 2010) explains this phenomenon by assuming that memories of traumatic events are stored in a highly associative network structure, which can be modified by experiences. After the initial experience of a traumatic event, subsequent traumatic events merge in the same network and it becomes increasingly difficult to correctly remember the corresponding contextual information of a specific traumatic event. Due to these memory deficits, one would expect difficulties in correctly remembering the number of traumatic events experienced. Moreover, retrospective reports of the frequency of particular events, as well as the age an event happened, might be even more severly impaired. Indeed, there is some evidence for an increase in the reported frequency (Roemer et al., 1998) and number (Southwick, Morgan, Nicolaou, & Charney, 1997) of traumatic events over time, and this amplification was significantly predicted by PTSD symptom severity. However, another study did not observe any systematic change in reported traumatic events over time (Bramsen et al., 2001). Given this inconsistency of the literature, it is of interest to investigate whether retrospective reports of trauma exposure are stable over time and if time stability varies as a function of PTSD diagnosis.

## Objective

Given the literature, there is an evident need to further evaluate the reliability as well as the predictive validity of reported traumatic events. In a sample of survivors of the conflict between the rebel group Lord's Resistance Army (LRA), and the Ugandan government, this study aimed at investigating (1) whether it is possible to reliably assess the event frequency and the age at trauma exposure in addition to the number of different event types experienced and (2) whether this additional assessment improves the prediction of PTSD risk.

## Methods

### Participants

Study participants were interviewed in villages of Nwoya district, Northern Uganda, an area that was severely affected by the LRA war. Participants survived and witnessed numerous traumatic events including abductions and forced recruitment of children and adolescents by the LRA, killings, mutilations, and sexual violence.

The study procedures were initially introduced to the villagers in community meetings, where we explained the aim and the scope of the research project. Community members who were interested in participating were invited to approach us to schedule an appointment. We recruited 240 participants into the present study. Inclusion criteria were (1) age between 18 and 65, (2) a history of trauma exposure, (3) absence of psychotic symptoms, and (4) absence of signs of alcohol addiction. Based on the detailed discussions with the interviewers and the examination of interview protocols, 13 individuals had to be excluded from the analyses for signs of current alcohol abuse (*N*=10), a history of psychotic symptoms (*N*=1), and difficulties in understanding interview questions (*N*=2), resulting in a sample of (*N*=227) (54% female, mean age=33.30, SD=10.56). After a detailed explanation of the study protocol, participants gave written inormed consent. All procedures followed the *Declaration of Helsinki* and were approved by the institutional review board of Gulu University, Uganda, the Ugandan National Council for Science and Technology (UNCST), and the ethics committee of the German Psychological Society (Deutsche Gesellschaft für Psychologie, DGPs).

### Procedure

Trained local interviewers performed the diagnostic interviews under the supervision of psychologists specialized in psychotraumatology. The interviewers attended a 6-week training on the concepts of mental health disorders, trauma and PTSD, counseling skills, and quantitative data collection. All study instruments were translated into the local language, Luo. Translations were followed by blind back-translations into English and group discussions with independent interpreters to ensure a valid translation of the instruments.

A 62-item event list was employed to assess trauma exposure. This event list included common traumatic experiences (e.g., natural disasters, accidents) that are also part of general traumatic event lists (e.g., life events checklist; Gray et al., 2004) but additionally included several war-related traumatic events (e.g., being close to a bomb attack), as well as events specific for the LRA conflict (e.g., being forced to eat human flesh).

For each event, participants were initially asked if this event ever happened to them. We calculated the *number of traumatic event types experienced* as the number of the affirmative answers. For each experienced event type, participants were further interviewed about the frequency of the respective event and their age at the time of the event. The event frequency was assessed in the categories never, 1 time, 2–3 times, 4–10 times, and ≥11 times. The categories were chosen in order to account for observed difficulties in remembering the exact event frequencies in the case of high trauma exposure. The event frequencies were scored on a Likert scale ranging from 0 (never) to 4 (≥11 times). The *Experienced Traumatic Events Frequency Score* was calculated as the sum score of these frequency values. Accordingly, when interpreting the score, one must be aware that it represents a frequency estimate rather than a score reflecting the exact frequency values.

Finally, respondents were asked about their age at the time a particular event happened to them. If an event was experienced more than once, multiple answers were possible. Age was assessed in the categories <6, 6–13, and ≥14. We calculated the number of events that happened in each age category, resulting in the variables *number of experienced traumatic event types under the age of 6*, *number of experienced traumatic event types under the age of 14*, and *number of experienced traumatic event types as an adult*. Supplementary Table 1 illustrates the traumatic event assessment for two example items.

After having completed the event list to assess traumatic events exposure, the same interviewer conducted a diagnostic interview based on the Posttraumatic Diagnostic Scale (PDS; Foa, 1995) to diagnose current and lifetime PTSD according to DSM-IV, as well as current PTSD symptom severity. The reliability and validity of the translated PDS has been assured in a prior investigation (Ertl et al., 2010). In total, 50 individuals (22%) fulfilled the diagnosis of current PTSD, and 163 (72%) fulfilled the criteria of a lifetime diagnosis of PTSD. Accordingly, 113 individuals (50%) had a history of PTSD but no current PTSD (i.e., remitted PTSD), and 64 (28%) never met the diagnostic criteria for PTSD.

The reliability analyses were performed in a subset of this sample (*N*=56) to whom we administered the event list twice, with a test–retest interval of 1 week. Participants for the reliability analyses were selected based on the information from the initial interview regarding two criteria: (1) We intended to obtain an equal number of participants in the three diagnostic groups (i.e., current, remitted, and never PTSD). (2) The diagnostic groups were matched by age, sex and education. For the purpose of an independent validation, respondents were assigned a different interviewer for the second interview.

### Statistics

All statistical analyses were performed in the statistical environment R version 3.1.0. (R Core Team, 2014).

#### Reliability analyses

Demographic and clinical data of the reliability sample was analyzed by ANOVA *F-*tests for continuous data if model residuals were normally distributed and the corresponding non-parametric test (i.e., Kruskal–Wallis *H-*test) if residuals were non-normally distributed. To analyze categorical data, we employed Fisher's Exact Test. If the omnibus *F*-test or Kruskal–Wallis *H*-test indicated significant group differences, we calculated *post hoc* tests with corrections for multiple comparisons to further examine which means differed significantly. Tukey's honestly significant difference was calculated as a parametric *post hoc* test, and the multiple comparison test after Kruskal–Wallis (Giraudoux, 2014; Siegel & Castellan, 1988) was employed as a non-parametric *post hoc* test.

We next calculated Pearson correlations between the first and second assessment to estimate the test–retest reliability of the respective trauma measures. To compare the derived reliability coefficients, differences in Pearson correlation coefficients were tested using the procedures for comparing non-overlapping correlations from the same sample (Raghunathan, Rosenthal, & Rubin, 1996) implemented in the R package cocor 1.0–1 (Diedenhofen & Musch, 2015). To compare the stability of the trauma measures per diagnostic group, we fitted linear mixed effect models utilizing the R package nlme 3.1–117 (Pinheiro, Bates, DebRoy, Sarkar, & The R Development Core Team, 2013). The respective trauma measurement was defined as the outcome variable, group as a between person fixed factor, time of measurement as a within-person fixed factor, and participants as a random effect.

#### Predictive validity analyses

To compare the different trauma exposure measurements regarding their ability to measure the construct trauma load as a risk factor for PTSD, we assessed the predictive validity of the respective measures by evaluating their ability to predict PTSD. Since our assessment took place 8 years after the end of the LRA war, the primary variable of interest was lifetime PTSD. In addition, we investigated the relationship between the trauma exposure measures and current PTSD, as well as current PTSD symptom severity.

We fitted logistic regression models to evaluate the influence of the trauma exposure measures on the binary outcome variables of lifetime and current PTSD. Regarding the continuous outcome of PTSD symptom severity, we initially fitted linear regression models; however, due to an excess of small values at low-levels of trauma exposure, assumptions regarding normal distribution of residuals and homoscedasticity were violated. Therefore, we fitted negative binomial regression models as recommended for overdispersed data (Hilbe, 2011). While negative binomial regression models for the various trauma measures on PTSD symptomatology generally revealed good model fits, they had the disadvantage of predicting an exponential increase of PTSD symptoms, which led to an unrealistic rise especially at high levels of traumatic load. A psychologically more plausible model was obtained by modeling trauma load with cubic splines (Harrell, 2001), with one knot set at the median of the respective trauma measurement. It is important to note that the hierarchy of the various trauma measures regarding their ability to predict PTSD symptomatology did not change as a function of the statistical model chosen to fit the data.

The ability of the different trauma exposure measures to predict PTSD risk and symptomatology was compared by estimating Akaike's Information Criterion (AIC) for each fitted model, as recommended by Burnham and Anderson (2002). In addition, the pseudo-*R* ^2^ statistic Nagelkerke's *R* ^2^ was estimated for the negative binomial and the logistic regression model as a measurement of explanatory power. For the logistic regression models, it is further feasible to calculate the coefficient of discrimination (D), which summarizes the ability of a model to discriminate between the two possible outcomes of a binary variable and was recommended as a measure of explanatory power. Analogous to the coefficient of determination (*R* ^2^), D can also vary between 0 and 1 (Tjur, 2009).

Statistical significance was determined by comparing nested models (i.e., models including the respective trauma variable vs. models excluding it) by means of likelihood ratio (LR) tests (Harrell, 2001). LR tests have the advantage that they can be calculated for both negative binomial regression and logistic regression models. The resulting test statistics approximates a *χ* ^2^ distribution and can hence be tested for significance by a *χ* ^2^ test. In order to account for potential violations of distributional assumptions, we additionally determined statistical significance non-parametrically by permutation tests using 10,000 random permutations. As the derived *p*-values did not differ between the two approaches, parametric *p*-values are reported. The stability of the fitted dose–response curves of cumulative trauma exposure on the outcome variables, which are depicted in Fig. 2, was assessed via 10,000 repeats of bootstrapping of the fitted values.

## Results

### Reliability

The reliability analyses are based on the subsample (*N*=56). The three diagnostic groups did not differ in gender distribution, age, and education. As expected, significant differences were observed in the trauma variables except for the number of traumatic events experienced under the age of 14. As only five individuals reported trauma exposure under the age of 6, the low number of observations prevented further analyses of this variable. Furthermore, the diagnostic groups differed in PTSD symptom severity (Table 1).

**Table 1:** Demographic and clinical information by diagnostic group

	Current PTSD (*N*=19)		Remitted PTSD (*N*=18)		Never PTSD (*N*=19)	Statistic^a^	*p*
*N* female (%)	9 (47)		9 (50)		10 (53)	Fisher's exact test	1.00
Mean age (SD)	34 (8.88)		35.56 (12.20)		33.95 (10.44)	*F*_2.53_=0.14	0.872
Mean number of school years (SD)	5.37 (2.52)		6.28 (2.91)		5.87 (3.70)	*F*_2.53_=0.40	0.670
Mean number of event types lifetime (SD)	37.58 (8.75)^b^	>	28.72 (6.05)	=	22.05 (8.13)	*H*_2_=24.01	<0.001
Mean experienced events frequency score lifetime (SD)	79.00 (21.12)^b^	>	46.78 (14.12)	=	39.37 (21.74)	*F*_2.53_=22.35	<0.001
Mean number of event types experienced under the age of 6 (SD)	0.05 (0.23)	=	0.28 (0.57)	=	0.00 (0.00)	*H*_2_=6.05	0.049
Mean number of event types experienced under the age of 14 (SD)	4.26 (8.88)		6.22 (8.52)		3.32 (4.63)	*H*_2_=0.54	0.762
Mean number of event types experienced as an adult (SD)	33.74 (9.91)^b^	>	23.83 (8.38)	=	19.16 (8.21)	*F*_2.53_=13.35	<0.001
Mean PDS score (SD)	14.89 (4.99)^b^	>	1.67 (1.68)	=	1.21 (1.99)	*H*_2_=38.67	<0.001

PDS, Posttraumatic Diagnostic Scale.

a ANOVA *F*-test for continuous data if test residuals were normally distributed, Kruskal–Wallis *H*-test for continuous data if residuals were not normally distributed, and Fisher's exact test for categorical data. Global comparisons of the means of continuous variables for the three groups were followed by parametric or non-parametric *post hoc* tests, if the *F*-test or Kruskal–Wallis *H*-test was significant. The results of the *post hoc* tests are visualized by the symbols >, <, and =.

b Indicates a significant difference between the current PTSD and never PTSD group.

**Fig. 1:**
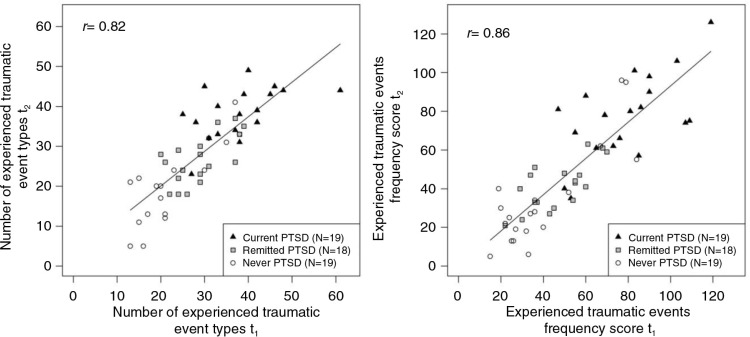
Test–retest reliability of trauma exposure assessed as the number of different traumatic event types experienced (left) and assessed as the experienced traumatic events frequency score (right). The line represents the linear relationship (estimated by fitting an ordinary least square regression) between the two repeated measurements across the entire reliability sample.

All trauma measures yielded high test–retest reliabilities (number of traumatic event types experienced, *r*=0.82, Fig. 1; experienced traumatic events frequency score, *r*=0.86, Fig. 1; number of experienced traumatic event types under the age of 14, *r*=0.82; and the number of experienced traumatic event types as an adult, *r*=0.83). There were no statistically significant differences between these four correlation coefficients (all *p*>0.2).

To investigate the stability of the trauma report and identify potential differences in the consistency of the reports as a function of diagnostic group, we calculated linear mixed effect models with the trauma exposure variables as the outcome variable. These models generally neither revealed a significant main effect of time, which would indicate a systematic change in the reports, nor an interaction diagnostic group×time for all investigated trauma exposure measures. The only exception was the *Experienced Traumatic Events Frequency Score*, which showed a slight decrease over time (*F*_1,53_=4.26, *p*=0.04), but no diagnostic group×time interaction effect. Supplementary Figs. 1 and 2 illustrate these analyses.

### Predictive validity

**Fig. 2:**
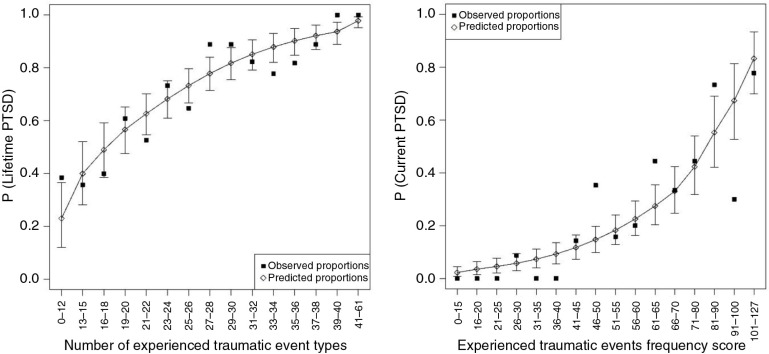
Left panel: The probability of lifetime PTSD is best predicted by a trauma assessment which measures the number of different traumatic event types experienced. Depicted are the observed and predicted proportions of lifetime PTSD against the number of traumatic event types, with 95% bootstrapped confidence intervals of the prediction. Right panel: By contrast, the probability of current PTSD is best predicted by a trauma assessment which considers the frequency of the traumatic events experienced. Depicted are the observed and predicted proportions of current PTSD against the experienced traumatic events frequency score, with 95% bootstrapped confidence intervals of the prediction. For this graphical illustration, data on trauma exposure was aggregated in groups of ≥9 individuals per group in order to be able to calculate meaningful proportions.

The predictive validity analyses are based on the entire sample (*N*=227). For the prediction of lifetime PTSD, a model including the *number of traumatic event types experienced* as a trauma measurement yielded the smallest AIC and was therefore chosen. Table 2 provides a summary of the goodness of fit statistics for the prediction of lifetime PTSD, current PTSD, and current PTSD symptom severity. The *number of traumatic event types* strongly predicts the risk of lifetime PTSD in a dose-dependent manner (Fig. 2, LR=43.88, *p*<0.00001). A quite good prediction of lifetime PTSD was also possible from the *Experienced Traumatic Events Frequency Score* (LR=31.81, *p*<0.00001), whereas the prediction from childhood or adult events alone was much weaker.

**Table 2:** Goodness of fit statistics for models including different trauma measures as predictors of lifetime PTSD, current PTSD, and current PTSD symptom severity

	AIC	D	Nagelkerke's *R* ^2^
Prediction of lifetime PTSD			
Number of traumatic event types experienced	230.15	0.19	0.25
Experienced traumatic events frequency score	242.22	0.13	0.19
Number of experienced traumatic event types under the age of 14	270.42	0.01	0.02
Number of experienced traumatic event types as an adult	252.06	0.09	0.13
Prediction of current PTSD			
Number of traumatic event types experienced	193.09	0.22	0.30
Experienced traumatic events frequency score	186.91	0.25	0.34
Number of experienced traumatic event types under the age of 14	238.92	0.02	0.03
Number of experienced traumatic event types as an adult	225.79	0.08	0.11
Prediction of current PTSD symptom severity			
Number of traumatic event types experienced	1103.87	–	0.31
Experienced traumatic events frequency score	1100.28	–	0.33
Number of experienced traumatic event types under the age of 14	1148.89	–	0.04
Number of experienced traumatic event types as an adult	1141.75	–	0.08

AIC, Akaike's Information Criterion; D, Coefficient of discrimination. Displayed is the goodness of fit statistic from regression models with the different trauma measurements as predictors. For the prediction of lifetime and current PTSD, logistic regression models were fitted, whereas a negative binomial regression with cubic splines was estimated for the prediction of current PTSD symptom severity.

By contrast, the risk of developing current PTSD was best predicted by the score considering event frequencies (Fig. 2, LR=56.46, *p*<0.00001; see Table 2 for a summary of the model selection procedure). Yet, the *number of traumatic event types experienced* was also a valid predictor of current PTSD (LR=50.28, *p*<0.00001).

Similarly, the model including the *Experienced Traumatic Events Frequency Score* yielded the best model fit for current symptom severity (Supplementary Fig. 3, LR=54.37, *p*<0.00001; see Table 2 for the model selection procedure). A good prediction of current symptom severity was also obtained from a model including the *number of traumatic event types experienced* as a trauma measurement (LR=50.78, *p*<0.00001). Again, the prediction of current PTSD risk and symptomatology from childhood or adult traumatic events alone led to a worse model fit.

## Discussion

### Reliability of trauma assessment

All trauma measures yielded good test–retest reliability, and reliability coefficients did not differ significantly. Hence, in contrast to previous concerns regarding the reliability of self-reported trauma frequencies (Neuner et al., 2004; Roemer et al., 1998), this study indicates that it is possible to reliably assess an event frequency score (based on frequency categories) in a severely traumatized post-conflict population. Furthermore, traumatic events experienced in the age range of 6–13 were also reliably reported, while events under the age of 6 were so rare that a reliability assessment was impossible. Therefore, the results of this study imply that a complex trauma assessment is feasible and yields reliable measurements, even in a highly traumatized population with little access to education.

### Predictive validity of trauma assessment

We replicated the *building block effect* of the number of traumatic event types experienced on PTSD risk and symptomatology (Kolassa et al., 2010; Mollica et al., 1998; Neugebauer et al., 2009; Neuner et al., 2004) and extended this finding by showing that a similar dose–response effect exists if traumatic event frequencies are additionally considered.

Concerning the prediction of lifetime PTSD risk, the additional assessment of event frequencies did not enhance prediction accuracy. By contrast, model fit was much better in a model including the simpler and less time-consuming assessment that measures the number of traumatic event types experienced. Hence, for the prediction of lifetime PTSD, which is the most important variable in cross-sectional etiological research, the number of traumatic event types experienced would be the variable of choice.

With respect to the diagnosis of current PTSD and current PTSD symptom severity, however, prediction was slightly better from a model including traumatic event frequencies. Two different interpretations may explain this finding. On the one hand, the repeated exposure to similar traumatic events could additionally strengthen the fear memories and lead to strong and long-lasting PTSD symptoms. On the other hand, it would be also possible that current PTSD symptoms bias the retrospective recollection of the frequency of traumatic experiences (Roemer et al., 1998). Individuals who frequently experience intrusions of traumatic events may retrospectively overestimate their frequency which would also lead to a strong relationship between current (but not lifetime) PTSD and reported event frequencies.

### No effect of developmental timing in this sample

Surprisingly, we did not observe a pronounced effect of childhood trauma on PTSD risk and symptomatology. Several explanations might account for this effect. First, rates of early trauma were quite low in this sample, and the majority of traumatic events in the context of LRA abductions were experienced during adolescence or adulthood. Second, the severe atrocities committed by the LRA may have such a strong impact that timing of traumatization did not matter. For instance, unpublished data from a different sample of young adults in Northern Uganda also indicates that the developmental timing of the LRA abduction did not influence PTSD risk (Anett Pfeiffer, personal communication). This is in line with accumulating evidence that repeated interpersonal trauma exposure in adulthood (e.g., torture) can lead to similar complex trauma reactions as childhood traumatization (McDonnell, Robjant, & Katona, 2013). Third, there is evidence that childhood trauma is stronger associated with symptoms of depression than with PTSD (Rieder & Elbert, 2013). Similarly, an investigation of 1,966 German women indicated that the conditional risk to develop PTSD after a traumatic event was equal for childhood and adult traumatization, while the risk to develop depression was more pronounced after childhood trauma (Maercker, Michael, Fehm, Becker, & Margraf, 2004). Further studies from conflict and peaceful societies with greater variability in childhood trauma exposure are needed to better understand the psychological risks associated with early traumatization.

### Study limitations and future research directions

The test–retest interval for the reliability analyses was relatively short, and future research should investigate whether the retrospective reports remain stable over longer time periods. Furthermore, similar to other studies measuring traumatic event frequency (Keane et al., 1989; Roemer et al., 1998; Unger et al., 1998), we assessed traumatic event frequency in categories as opposed to exact event frequencies. This decision was made since we observed that individuals with high trauma exposure had difficulties recalling the exact event frequencies but were able to provide categorical answers. Hence, the reliability of exact event frequencies will have to be addressed in subsequent studies investigating individuals with lower trauma exposure.

Finally, our results were obtained from a very specific population of LRA war survivors, and it has to be investigated whether the results can be replicated in independent populations. The majority of study participants (62%) have been abducted by the LRA, and/ or had to leave their home during the war to seek protection (93%). Hence, next to the repeated traumatic experiences, this population was also exposed to several chronic stressors. Therefore, the frequency of traumatic experiences or childhood trauma exposure might have a stronger impact on PTSD risk in different settings and under conditions of less extreme traumatization and chronic stress.

## Conclusions

The results of this study indicate that the assessment of event types, as well as an additional evaluation of event frequencies, yields reliable and valid trauma measurements. Considering lifetime PTSD, which is the most interesting variable in the investigation of risk factors for PTSD development, the classical trauma exposure variable (i.e., the number of traumatic event types experienced) leads to the best prediction. As a detailed recollection of traumatic experiences is stressful especially for survivors suffering from PTSD (O’Kearney & Parry, 2014), an assessment considering event frequencies might inflict unnecessary levels of stress on trauma survivors as it requires the participants to recall the different times the event happened in order to give a frequency estimate. By contrast, the assessment of types only requires the response “yes” or “no” from the participant and does not further encourage reflection about the different times the respective event happened. Furthermore, the assessment of event types as opposed to frequencies is less time-consuming and hence represents the more economical way to assess trauma exposure if resources are limited. Taken together, we would therefore recommend the number of traumatic event types experienced as a reliable, valid, and relatively less strenuous measurement for the assessment of cumulative trauma exposure.
